# Association of TRPV1 and the SIRT3/SOD2 Signaling Pathway in Mononuclear Cells and Astrocyte-Derived Extracellular Vesicles in Patients with Schizophrenia

**DOI:** 10.3390/brainsci15040339

**Published:** 2025-03-25

**Authors:** Rui Xu, Hao Liu, Chang Shu, Yuan Li, Shijing Wang, Ying Xiong, Fashuai Chen, Xiaowei Wang, Huan Huang, Zhongchun Liu, Gaohua Wang, Huiling Wang

**Affiliations:** 1 Department of Psychiatry, Renmin Hospital of Wuhan University, Wuhan 430060, China; 2022283020148@whu.edu.cn (R.X.); tmliuhao@whu.edu.cn (H.L.); rm001522@whu.edu.cn (C.S.); 2016302180200@whu.edu.cn (Y.L.); shijingwang@whu.edu.cn (S.W.); 2022203020025@whu.edu.cn (Y.X.); 2023103020033@whu.edu.cn (F.C.); 2023283020109@whu.edu.cn (X.W.); rm003229@whu.edu.cn (H.H.); zcliu6@whu.edu.cn (Z.L.); wgh6402@whu.edu.cn (G.W.); 2 College of Life Sciences, Wuhan University, Wuhan 430072, China; 3 Hubei Institute of Neurology and Psychiatry Research, Wuhan 430060, China; 4 Hubei Provincial Key Laboratory of Developmentally Originated Disease, Wuhan 430071, China

**Keywords:** schizophrenia, TRPV1, extracellular vesicles, PBMC, Sirt3/SOD2 signaling pathway

## Abstract

Objectives: The transient receptor potential vanilloid type 1 (TRPV1) is a factor that mediates glial cell response with effects on mitochondrial function. It may affect the occurrence and development of schizophrenia. The aim of this study is to further explore schizophrenia biomarkers by analyzing TRPV1 and oxidative stress in astrocyte-derived extracellular vesicles (ADEs) and peripheral blood mononuclear cells (PBMCs). Methods: A case–control study was conducted. The Positive and Negative Syndrome Scale and the Brief Assessment of Cognition in Schizophrenia (BACS) clinical data were obtained from 50 symptomatic patients with schizophrenia and 50 controls, and fasting peripheral blood samples were collected for the isolation of PBMCs and ADEs. Western blotting was used to assess TRPV1, Sirtuin3 (Sirt3), SOD2, and acetyl-SOD2. Results: The patient group exhibited significantly reduced TRPV1 and Sirt3 expression levels in PBMCs and ADEs compared with the control group. In addition, there was a marked increase in SOD2 and acetyl-SOD2 levels. TRPV1 was negatively correlated with the negative symptom score in the patient PBMCs and ADEs. SOD2 showed positive correlations with the general psychopathology symptom score, and acetyl-SOD2 was positively correlated with the negative symptom score. The BACS total score was positively correlated with TRPV1 levels and negatively correlated with acetyl-SOD2 levels in the patient group. Conclusion: TRPV1 expressions in PBMCs and ADEs were reduced and closely correlated, and TRPV1 levels were associated with psychiatric symptoms and cognitive function in patients with schizophrenia. It was indicated that TRPV1 could be a biomarker for schizophrenia and reflect the disease severity.

## 1. Introduction

Schizophrenia is one of the most severe psychiatric disorders and is characterized by its chronic nature, high prevalence, significant disability rates, and limited treatment efficacy [1]. It contributes to the overall disease burden and places within the top ten conditions by this metric. The primary schizophrenia manifestations include positive symptoms (hallucinations, delusions, etc.), negative symptoms (affective flattening, anhedonia, etc.), and cognitive deficits (executive functioning impairments, working memory deficits, etc.). The current schizophrenia diagnostic practices predominantly rely on clinical assessments that incorporate the patient’s psychiatric history and mental status evaluations [2], as no reliable biomarkers exist for supplementary diagnosis [3]. Although the pathophysiological mechanisms that underlie schizophrenia remain incompletely understood, numerous studies have indicated that factors such as oxidative stress, neuroinflammation, dysregulation of neurotransmitters, neuronal apoptosis, and synaptic damage play substantial roles in its etiology [4].

The transient receptor potential vanilloid type 1 (TRPV1) channel is a ligand-gated, non-selective cation channel prevalent in the central nervous system (CNS) [5,6]. This receptor can be activated by various exogenous stimuli (including temperatures that exceed 43 °C, low pH, capsaicin, vanillic acid, and other biotoxins) as well as endogenous ligands (e.g., anandamide and lipoxygenase products) [7]. TRPV1 is involved in diverse pathophysiological processes that include oxidative stress, neuroinflammation, neuronal apoptosis, and synaptic plasticity alterations, and it may be a factor in the onset and progression of neuropsychiatric disorders [8,9,10,11,12]. A previous study found a decrease in TRPV1 expression in a schizophrenic rat model [11], and similar findings have been found in patients with schizophrenia [9].

The current evidence suggests that TRPV1 is expressed in glial cells, particularly in microglia and astrocytes [13]. TRPV1 influences glial cell responses, such as the secretion of inflammatory mediators and reactive oxygen species (ROS), by facilitating calcium ion influx [13]. Astrocytes are a principal type of glial cell, and they are vital for maintaining synaptic integrity, nerve fiber functionality, neurogenesis, and metabolic support. They also significantly contribute to neuroinflammatory processes, oxidative stress responses, and immune regulation [14]. Astrocytes can transition into a reactive state upon stimulation and produce pro-inflammatory mediators and promote ROS generation, thereby modulating inflammatory responses and potentially leading to neuronal degeneration or damage.

Sirtuin3 (Sirt3) is a member of the evolutionarily conserved nicotinamide adenine dinucleotide (NAD+)-dependent protein deacetylase family. It is predominantly located in mitochondria and exhibits robust deacetylase activity. It regulates oxidative stress levels by enhancing superoxide dismutase (SOD2) activity [15]. SOD2 is mainly located in mitochondria and is responsible for catalyzing the dismutation reaction of superoxide anion, converting it to hydrogen peroxide and oxygen. Research has demonstrated that Sirt3 plays a protective role against glial cell hyperactivation, oxidative stress, and neuroinflammation [16]. However, TRPV1 activation leads to an increase in intracellular calcium concentration, which in turn affects mitochondrial function [13]. Therefore, although the functions of TRPV1 and Sirt3 in cells are different, they are closely related through calcium ion regulation, mitochondrial function, and oxidative stress.

Recent studies have identified that astrocytes play an important role in the pathogenesis of schizophrenia; however, methodological constraints have posed significant challenges in the exploration of astrocytes in human subjects [17]. Extracellular vesicle extraction technique advancements now allow for in vivo assessments of CNS states without the need for brain biopsies [18]. Different extraction methods enable extracellular vesicle isolation from various sources, including astrocyte-derived extracellular vesicles (ADEs) [19]. CNS-derived extracellular vesicles can traverse the blood–brain barrier and can be obtained from the peripheral blood. The diverse molecular content of these extracellular vesicles reflects the physiological states of their originating cells.

There was a temporary deficiency in the investigation of the alterations in TRPV1 expression and oxidative stress levels within both central and peripheral systems in patients with schizophrenia. We speculate that the changes in TRPV1 expression in the central and peripheral systems are consistent and are correlated with oxidative stress levels. Therefore, the primary aim of this study is to examine alterations in TRPV1 expression and oxidative stress levels in ADEs and peripheral blood mononuclear cells (PBMCs) from patients with schizophrenia. In addition, exploratory analyses are conducted to (1) investigate the correlation between TRPV1 and oxidative stress-related markers in ADEs and PBMCs from patients with schizophrenia and (2) assess the relationship between these biomarkers and clinical characteristics.

## 2. Methods

### 2.1. Participants

Inclusion criteria of the patient group: (1) participants must have met the diagnostic criteria for schizophrenia as outlined in the *Diagnostic and Statistical Manual of Mental Disorders, Fifth Edition* (DSM-V), and have been assessed by two attending psychiatrists (Huiling Wang and Hao Liu); (2) they must have achieved a total score of 60 or higher on the Positive and Negative Syndrome Scale (PANSS); (3) they should have been between the ages of 18 and 45; (4) they must have provided informed consent, demonstrated cooperation during assessments, and been capable of completing both clinical evaluations and the collection of peripheral blood samples.

Inclusion criteria of the control group: (1) participants who were assessed by two attending psychiatrists (Huiling Wang and Hao Liu) and had no prior history of mental illness or familial history of such disorders; (2) they should have been between the ages of 18 and 45; (3) they must have provided informed consent, demonstrated cooperation during assessments, and been capable of completing both clinical evaluations and the collection of peripheral blood samples.

Exclusion criteria: (1) individuals who were diagnosed with organic brain disorders, had coexisting mental health issues, or suffered from severe physical illnesses; (2) those who declined to take part in this study; (3) participants who could not understand the assessment procedures or demonstrated impulsive aggression; (4) women who were pregnant or breastfeeding; (5) individuals with a history of substance abuse, smoking, alcohol use, or consumption of irritating foods (like spicy peppers and garlic) within one week prior to blood sampling.

A total of 54 healthy controls and 52 individuals diagnosed with schizophrenia were initially recruited for this research. Following the exclusion criteria, 50 subjects were retained in both the control and patient cohorts, and they were matched for age and sex. The healthy control participants were sourced from local educational institutions and community settings, while the schizophrenia cohort was obtained from outpatient and inpatient populations at Renmin Hospital of Wuhan University. This research was conducted between September 2023 and June 2024. Ethical approval was obtained from the Ethics Committee of Renmin Hospital of Wuhan University (WDRY2023-K141), and informed consent was secured from the participants or their legal representatives. This research has also been duly registered in the Chinese Clinical Trial Registry (ChiCTR2400090643).

### 2.2. Clinical Data Collection

Demographic and clinical data collection: Data pertaining to gender, age, height, weight, and educational attainment of all participants were recorded. In addition, information on the episode frequency, disease progression, pharmacological treatments, and familial histories of the patients with schizophrenia was gathered.

Scale assessments: (1) The Positive and Negative Syndrome Scale (PANSS) [20]: The PANSS is extensively utilized in clinical settings to evaluate patient psychotic symptom severity. It comprises three components: the positive symptom subscale, the negative symptom subscale, and the general psychopathology subscale. Higher total and subscale scores indicate a more severe condition. (2) Brief Assessment of Cognition in Schizophrenia (BACS) [21]: This scale assesses cognitive function across six domains that include verbal memory, working memory, processing speed, verbal fluency, attention, and executive function. Higher scores reflect superior cognitive performance in subjects.

### 2.3. Extraction of PBMCs

Morning blood samples were collected, and then the PBMCs were isolated following the protocol provided by the lymphocyte separation medium (Absin, cat. no. abs930). A volume of 2 mL of whole blood was drawn into a sterile, enzyme-free 15 mL conical centrifuge tube, followed by the addition of 2 mL of phosphate-buffered saline (PBS), ensuring thorough mixing. Subsequently, 3 mL of lymphocyte separation medium was placed into a new conical centrifuge tube, and 4 mL of the blood–PBS mixture was carefully layered on top of the lymphocyte separation medium while maintaining liquid stratification. The tube was positioned in a horizontal centrifuge without a centrifugal brake and spun at 300× *g* for 20 min. Upon completion of centrifugation, the liquid was segregated into four distinct layers: the diluted plasma layer, the peripheral blood mononuclear cell layer, the lymphocyte separation buffer layer, and the erythrocyte layer from top to bottom. The entire volume of the mononuclear cell layer was aspirated using a pipette and transferred to a new conical centrifuge tube. PBS was added to the dilute and thoroughly mixed. The samples were then centrifuged at 1500 rpm for 10 min, after which the supernatant was discarded. The resulting pellet was resuspended in an appropriate volume of PBS and transferred to a cryogenic storage tube. The samples underwent a second centrifugation at 1500 rpm for 10 min, and the supernatant was completely removed. The isolated PBMCs were then stored at −80 °C with the addition of 10 μL of phenyl methane sulfonyl fluoride (PMSF, Beyotime, Shanghai, China, cat. no. ST506).

### 2.4. Extraction of ADEs

The protocol of Jia L et al. for exosome extraction was referenced [22]. Frozen serum samples were gradually thawed at room temperature. A 250 μL serum volume was processed using the Exo Quick kit (System Biosciences, Wuhan, China, cat. no. EXOQ20A-1) to isolate the total exosome precipitate. This precipitate was resuspended in 350 μL of Dulbecco’s phosphate-buffered saline (DPBS) (Servicebio, Wuhan, China, cat. no. G4200) devoid of calcium and magnesium ions and with the addition of phosphatase and protease inhibitors (Beyotime, cat. no. P1046). Subsequently, 50 μL of 3% bovine serum albumin (BSA) (Beyotime, cat. no. ST023) was added. This mixture was then incubated with 2 μL of biotinylated glutamine–aspartate transporter antibody (Miltenyi Biotec, Bergesch Gladbach, Germany, cat. no. 130118984) for 1 h at room temperature. A total of 10 μL of streptavidin nucleic acid resin (Thermo Fisher Scientific, Massachusetts, America, cat. no. 53116) and 40 μL of 3% BSA were then added for an additional 30 min at room temperature. The resulting precipitate was resuspended in 100 μL of pre-cooled 0.05 M glycine–HCl buffer (pH = 3.0) and centrifuged at 4000× *g* for 10 min to discard the supernatant. The solution pH was adjusted to 7.0 by adding 25 μL of DPBS that contained 3% BSA and 10 μL of 1 M Tris–HCl (pH = 8.0, Beyotime, cat. no. ST780) to obtain the ADEs.

### 2.5. Western Blot

PBMC and ADE samples from the above 50 patients with schizophrenia and 50 controls were assayed for protein using Western blot. The protein concentration was quantified using the BCA assay kit (Beyotime, cat. no. P0012S) as per the manufacturer’s protocol. The samples were then mixed with protein loading buffer to achieve a protein concentration of 10 μg/μL and denatured by boiling for 10 min. Samples and Prestained Protein Marker II (Servicebio, cat. no. G2058) were loaded and electrophoresed with a loading volume of 2 μL per well and then transferred to the membrane. Blocking was performed with the protein-free rapid blocking solution (Servicebio, cat. no. G2052) for 5 min. Incubation was then performed with the primary antibody (TRPV1 1:1000, Abcam, Cambridge, Britain, cat. no. ab305299; Sirt3 1:1000, Abcam, cat. no. ab217319; SOD2 1:750, Servicebio, cat. no. GB111875; acetyl-SOD2 1:1000, Abcam, cat. no. ab137037) diluted to the specified ratio at 4 °C overnight. The membrane was thoroughly washed with Tris-buffered saline with Tween 20 (TBST) and then incubated with the rabbit-derived secondary antibody (1:10,000, Servicebio, cat. no. GB23303) at 37 °C for 2 h. After additional washes with TBST, a full reaction with freshly prepared ECL working solution was followed by imaging visualization, with exposure conditions adjusted according to luminescence intensity. The analysis was conducted using Image J (Version 1.54) software, and the optical density was quantified.

### 2.6. Identification of ADEs

(1) A morphological assessment of the ADEs was conducted using transmission electron microscopy (TEM). A 10 μL sample was placed on a copper grid after immunoprecipitation and incubated at room temperature for 5 min. This was followed by negative staining with a uranyl acetate solution for 1 min. Filter paper was used to remove the excess liquid, and the exosome morphology was examined and photographed under a TEM. (2) Nanoparticle tracking analysis: The size (nm) and concentration (particles/mL) of the ADEs were quantified using the ZetaView Particle Metrix (PMX-120, Particle Metrix, Meerbusch, Germany) instrument, and NTA 3.4.003 nanoparticle tracking software was used for the analysis. (3) Western blotting was performed to detect the three exosome markers: TSG101 (1:1000, Beyotime, cat. no. AF8259), CD81 (1:1000, Beyotime, cat. no. AG1530), and ALIX (1:1000, Beyotime, cat. no. AG4522).

### 2.7. Statistical Analysis

Variables that exhibited a normal distribution were presented as means ± standard deviations (±s), while those without a normal distribution were expressed using the interquartile range [M (P25, P75)]. An analysis of variance (ANOVA), chi-square tests, and independent sample *t*-tests were implemented to evaluate general demographic characteristics. An analysis of covariance was used to assess differences in biological markers across the groups. All biological indicators of the control group were standardized. The Pearson correlation coefficients facilitated an analysis of the relationships between variables. The data obtained were analyzed utilizing the Bonferroni adjustment. Statistical analyses and graphical representations were conducted using GraphPad Prism 9 software.

## 3. Results

### 3.1. Participant Characteristics

A total of 50 individuals diagnosed with schizophrenia and 50 matched healthy controls (HCs) were ultimately included in this study. The demographic characteristics and clinical profiles of both cohorts are shown in Table 1. No statistically significant differences were observed regarding gender, age, body mass index (BMI), or other variables. Nevertheless, the schizophrenia group exhibited a markedly reduced number of years of education compared with the HC group. The methodology established by Keefe RSE et al. [21] was used, and it was found that both the individual component and composite scores of the BACS were markedly lower in the schizophrenia group compared with the HC group.

### 3.2. Identification of Exosomes

**Figure 1:**
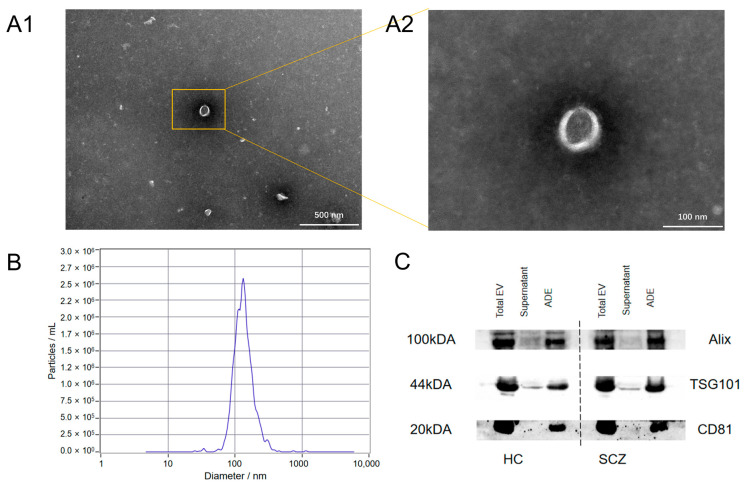
Identification of astrocyte-derived cellular exosomes: (**A**) shows the transmission electron microscope images of exosomes at different magnifications; (**B**) is the result of nanoparticle tracking analysis, showing the diameter distribution of astrocyte-derived extracellular vesicles (diameter: 128.8 ± 40.1 nm, detection concentration: 4.8 × 10^7^ particles/mL, original concentration: 9.5 × 10^11^ particles/mL); (**C**) shows exosome-specific protein Western blotting results. HCs: healthy controls; SCZ: schizophrenia; ADE: astrocyte-derived extracellular vesicle.

The extracted exosomes were cup-shaped with a near-circular central depression when viewed under a TEM (Figure 1A). They had diameters that ranged from 30 to 200 nm (Figure 1B). The Western blot results showed that the extracted exosome samples contained three exosome-specific proteins (CD81, TSG101, ALIX) (Figure 1C).

### 3.3. Differences in the TRPV1 Expressions and Partial Oxidative Stress Indicators in the PBMCs and ADEs

**Figure 2:**
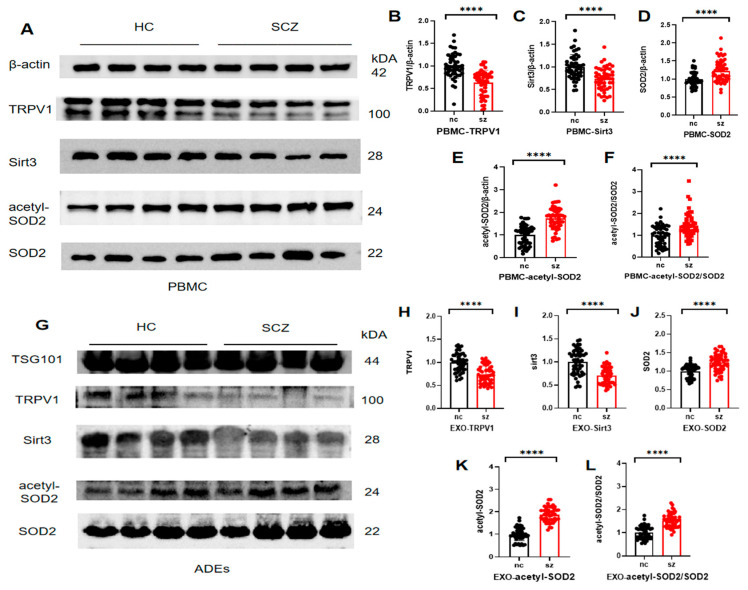
Expression of TRPV1 receptors and some markers of oxidative stress in PBMCs and ADEs in the control and patient groups: (**A**) is the result of Western blotting of PBMCs; (**G**) shows the results of Western blotting of ADEs; (**B**–**F**) shows protein expression and analysis in peripheral blood PBMCs; (**H**–**L**) shows the protein expression and analysis. The protein expression and ratio of the control group were homogenized, **** *p* < 0.0001. Abbreviations: HCs: healthy controls; SCZ: schizophrenia; PBMC: peripheral blood mononuclear cell; ADEs/EXO: astrocyte-derived extracellular vesicles; TRPV1: transient receptor potential vanilloid type 1.

The confounding variables, such as sex, age, and educational background, were adjusted, and the TRPV1 receptor and Sirt3 protein expressions in the PBMCs of the patients with schizophrenia were significantly diminished compared with those of the HC group. Conversely, the SOD2 and acetyl-SOD2 protein levels were markedly elevated in the patient cohort relative to those of the HC group. Furthermore, the acetyl-SOD2 to SOD2 ratio was significantly greater in the patient group than in the HC group, as illustrated in Figure 2A–F and Table S1.

The TRPV1 receptor and Sirt3 protein expressions in the ADEs were lower than those observed in the HC group, while the SOD2 and acetyl-SOD2 protein levels were elevated compared with those of the HC group. This result was consistent with the findings in the PBMCs. The acetyl-SOD2 to SOD2 ratio was also significantly increased in the patient group, as depicted in Figure 2G–L and Table S1.

### 3.4. Relationship Between TRPV1 and the Partial Oxidative Stress Indicators in the PBMCs and ADEs

**Figure 3:**
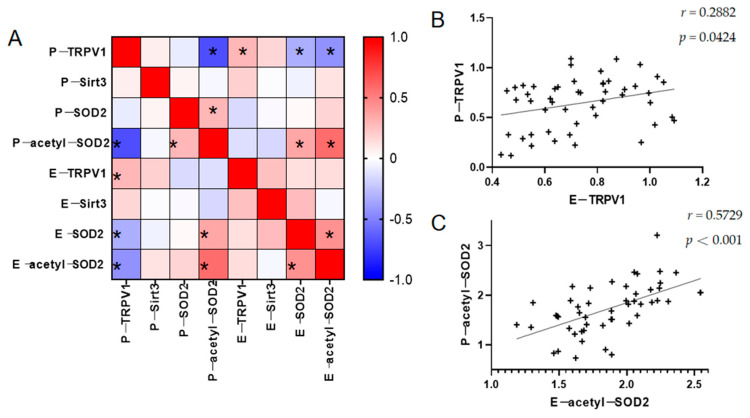
Relationship between TRPV1 and partial oxidative stress detection in PBMCs and ADEs from patients: (**A**) heat map of the expression differences of the above indicators; (**B**) correlation between TRPV1 expression level in ADEs and in PBMCs; (**C**) correlation between acetyl-SOD2 expression levels in ADEs and in PBMCs. * *p* < 0.05. Abbreviations: P-: peripheral blood mononuclear cell-; E-: astrocyte-derived extracellular vesicles-; TRPV1: transient receptor potential vanilloid type 1.

An analysis of TRPV1, Sirt3, SOD2, and acetyl-SOD2 in the PBMCs and ADEs of the patients with schizophrenia showed that TRPV1 in the PBMCs exhibited a positive correlation with that in the ADEs. Additionally, acetyl-SOD2 in the PBMCs was positively correlated with that in the ADEs, while the TRPV1 in the PBMCs showed a negative correlation with acetyl-SOD2. Furthermore, SOD2 was positively correlated with acetyl-SOD2, and SOD2 within the ADEs had a positive correlation with acetyl-SOD2. Refer to Figure 3 and Table S2 for further details. However, we only found a positive correlation between SOD2 and acetyl-SOD2 in both the PBMCs and ADEs in the HC group, as detailed in Table S3.

### 3.5. Relationship Between the TRPV1 Expressions and the Partial Oxidative Stress Indicators in the PBMCs and ADEs with Clinical Features

Confounding variables, such as sex, age, and educational attainment, were adjusted for, and the TRPV1 receptor expression levels in the PBMCs from the patients had a positive correlation with a positive symptom score and a negative correlation with a negative symptom score. In addition, Sirt3 expression had a negative correlation with a positive symptom score, while the SOD2 expression was positively associated with both the total score and the general pathological score. Furthermore, the acetyl-SOD2 expression showed a positive correlation with negative symptoms. The TRPV1 expression had a significant negative correlation with the presence of negative symptoms in the ADEs, and the Sirt3 expression had a significant negative correlation with positive symptoms, as illustrated in Table 2 and Table S4.

Upon adjusting for confounding variables, the BACS total score exhibited a positive correlation with the TRPV1 levels and a negative correlation with the acetyl-SOD2 levels in the PBMCs of the patient group. The BACS total score was positively correlated with the TRPV1 levels and inversely associated with both the SOD2 and acetyl-SOD2 levels in the ADEs of patients. A detailed analysis revealed that verbal memory, token motor, category fluency, word fluency, symbol coding, and the Tower of London were positively correlated with the TRPV1 expression in the PBMCs among patients. Conversely, verbal memory, token motor, category fluency, word fluency, and the Tower of London were negatively correlated with the acetyl-SOD2 expression in the PBMCs. Verbal memory and digit sequencing were positively correlated with the TRPV1 expression in the ADEs, while word fluency was negatively correlated with the SOD2 expression in the ADEs. Furthermore, category fluency, word fluency, and symbol coding were negatively correlated with the acetyl-SOD2 expression in the ADEs. Details are provided in Table 2 and Table S4. However, we found that the abovementioned indicators were much less correlated with BACS in the HC group, as illustrated in Table S5.

## 4. Discussion

Schizophrenia is a multifaceted and prevalent psychiatric disorder that exhibits substantial clinical heterogeneity and lacks definitive biomarkers, and its pathogenesis remains incompletely understood. In this study, we found a marked downregulation of Sirt3 and upregulation of SOD2 and acetyl-SOD2 in both the PBMCs and ADEs of patients with schizophrenia relative to the controls. Furthermore, the SOD2 and acetyl-SOD2 levels were associated with psychiatric symptoms and cognitive deficits. This result reinforced the role of oxidative stress in schizophrenia. Previous studies have also shown that oxidative stress levels are increased in schizophrenia [23,24,25]. Although oxidative stress is pivotal in neural damage, its precise mechanistic role in schizophrenia remains inadequately clarified. Oxidative stress, characterized by an imbalance in redox homeostasis, is intertwined with neuroinflammation. Both phenomena mutually exacerbate and modulate through various signaling pathways [26]. ROS are free radicals generated by mitochondrial activity during aerobic metabolism. They accumulate under redox disequilibrium and lead to cellular damage or apoptosis [27]. Antioxidants are categorized into enzymatic (e.g., superoxide dismutase and catalase) and non-enzymatic types (e.g., albumin, bilirubin, vitamin C, glutathione, and coenzyme Q). They mitigate this imbalance by neutralizing excess free radicals. SOD2 is an important antioxidant enzyme that eliminates free radicals produced in the mitochondria. However, SOD2 acetylation leads to a decrease in its activity and subsequently inhibits its action to resist oxidative stress. Mitochondrial Sirt3 is a potent deacetylase that modulates ROS production by regulating SOD2 and other antioxidant enzymes [28,29,30,31]. Numerous investigations have demonstrated that Sirt3 exhibits neuroprotective properties across various neurodegenerative disorders. This can be attributed to its proficient modulation of energy metabolism and oxidative stress responses [32,33,34]. In this study, Sirt3 was diminished in both the PBMCs and ADEs of patients with schizophrenia and negatively correlated with positive symptoms. This result suggested that Sirt3 also has a positive protective role in schizophrenia. Prior research conducted in our laboratory indicated that Sirt3 may play a role in reducing the schizophrenia-like behavioral phenotype at adulthood in a 24 h maternal separation Wistar rat model on postnatal day nine [16]. This result agrees with our clinical observations.

TRPV1 is a non-selective cation channel that is ubiquitous in mammals. It is implicated in various neuropsychiatric conditions that include anxiety [8], depression [7], schizophrenia [11], epilepsy [10], and Parkinson’s disease [12]. Its involvement may pertain to neuronal and glial activities such as synaptic plasticity, neurotransmitter release, and neuroinflammatory mediator secretion [35,36,37,38]. Our study revealed significantly lower TRPV1 levels in the PBMCs of the schizophrenic group compared with those of the HC group and a notable inverse correlation between TRPV1 and acetyl-SOD2 levels only in the patient group. Prior animal research demonstrated that TRPV1 activation in the substantia nigra can induce neurotrophic factor production and attenuate oxidative stress [39], while other studies have also shown that TRPV1 inhibition can reduce oxidative stress and neuronal damage [40]. The exact mechanism by which TRPV1 mediates oxidative stress remains unclear, and its dualistic role appears to depend on its distribution and the nature of the stimuli [37]. The abovementioned findings suggested that the pathological mechanism of TRPV1 in schizophrenia may be related to oxidative stress, which is also supported by the results of our study. In addition, the activation of TRPV1 results in an elevation of intracellular calcium levels, which can induce mitochondrial dysfunction and REDOX imbalance [40]. Meanwhile, Sirt3 modulates the activity of mitochondrial proteins via deacetylation, thereby preserving mitochondrial homeostasis [31]. In patients with schizophrenia, mitochondrial dysfunction leads to alterations in the activity of mitochondrial Sirt3 protein, exacerbating the accumulation of ROS and intensifying the clinical manifestations of schizophrenia.

As a crucial constituent of glial cells within the CNS, astrocytes are extensively distributed among neurons and play a pivotal role in sustaining the normal physiological functions of the CNS [41,42]. They are involved in providing metabolic support, enhancing communication between synapses, maintaining ion homeostasis, and scavenging free radicals, and they form an integral part of the blood–brain barrier [43,44,45,46]. Astrocyte mitochondria can modulate the redox state via the pentose phosphate pathway and glutathione metabolism, among other mechanisms. When astrocytes are activated, they may show dual effects; however, the predominant effect is detrimental, leading to redox imbalance, excessive ROS accumulation, oxidative stress, inflammatory responses, and mutual promotion of these processes [17]. ADEs are involved in communication between astrocytes and various cells and neurons, and the molecules (e.g., RNA, lipids, and proteins) that they carry regulate numerous physiological and pathological processes [47,48,49]. Moreover, ADEs can traverse the blood–brain barrier into the peripheral circulation. The molecular composition of ADEs varies with different states, and this provides a means to investigate the pathophysiological processes of CNS diseases. In this study, the detection of ADEs revealed that, similar to the findings in PBMCs, the TRPV1 and Sirt3 expressions were significantly downregulated, while the SOD2 and acetyl-SOD2 expressions were significantly increased in patients with schizophrenia compared with those of the HC group. This result indicated an oxidative stress change in the astrocytes of patients with schizophrenia.

A correlation analysis with the clinical symptoms indicated a negative association between TRPV1 expression in the PBMCs and ADEs of patients with schizophrenia and negative symptoms. We also found that TRPV1 was positively correlated with some subscales and the total BACS score in both the PBMCs and ADEs of the controls and the patients. However, the oxidative stress indexes, such as acetyl-SOD2, were negatively correlated with the cognitive assessment scale scores. These findings indicated that psychiatric manifestations and cognitive impairments in schizophrenia were associated with heightened oxidative stress levels and decreased TRPV1 levels. These results agreed with the results of prior bench studies, that is, the injection of a TRPV1 receptor antagonist in normal rats reduced social behavior and memory function [50]. Furthermore, the results of this study provided further evidence that implicated astrocytes in the pathogenesis and progression of schizophrenia and that they may be closely tied to the cognitive impairment of the disease [17,26,41,51].

This study unexpectedly revealed a positive association between TRPV1 and the positive symptoms. This could be potentially attributable to the interplay between TRPV1 and oxidative stress. Previous studies indicated that TRPV1 can regulate oxidative stress [52,53]. Concurrently, ROS generated during oxidative stress can directly activate TRPV1 [54], and the buildup of reactive nitrogen species can lead to increased TRPV1 expression [52]. In addition, inflammatory and ionic homeostasis dysregulation induced by oxidative stress may exert an indirect influence on TRPV1 activity [55]. These findings imply a bidirectional interaction between oxidative stress and TRPV1 activation. A meta-analysis that examined oxidative stress in patients with schizophrenia demonstrated significantly elevated levels of oxidative stress and inflammation during the acute phase compared with those in remission following treatment [25]. Our study also found that SOD2 and other oxidative stress markers were positively correlated with the positive symptoms in patients with acute onset schizophrenia. Therefore, we hypothesized that the imbalance of oxidative stress during this phase may trigger a partial activation of the TRPV1 protein. This would result in a transient increase in its expression. However, this feedback mechanism is insufficient to counteract the intrinsic downregulation of TRPV1 associated with schizophrenia progression.

Our study has several limitations. Due to the lack of fresh blood samples, other indicators of oxidative stress levels, such as free radicals, could not be detected. In addition, there is a significant interplay between oxidative stress and neuroinflammation, and further investigation into the subsequent changes in inflammatory mediators is required to elucidate potential mechanisms. Moreover, this study exclusively utilized a cross-sectional design, and this is insufficient to capture the roles of TRPV1 and oxidative stress responses in schizophrenia pathogenesis. Additionally, the distinction between first-episode and relapsed patients was not made, and this may have introduced bias in the analysis. Future studies will aim to incorporate a broader range of indicators, enhance patient stratification, and include longitudinal follow-ups for a more in-depth analysis.

## 5. Conclusions

TRPV1 expression was markedly reduced in patients with schizophrenia. Concurrently, TRPV1 expression in the PBMCs of the patient group exhibited a strong correlation with those in the ADEs and was associated with the clinical symptoms and cognitive impairments of schizophrenia. These findings corroborated the role of TRPV1 in the pathogenesis and progression of schizophrenia and indicated that TRPV1 could serve as a potential biomarker for schizophrenia and reflect schizophrenia severity.

## Figures and Tables

**Table 1:** Descriptive data of included participants.

Parameters	HC (*n* = 50)	SCZ (*n* = 50)	t/χ^2^/F	*p*
Sex (female/male)	25/25	24/26	0.198	0.843
Age (years)	24.09 ± 4.15	24.74 ± 6.07	1.391	0.167
Years of education (years)	16.61 ± 1.35	12.18 ± 3.35	7.911	<0.0001
BMI (kg/m^2^)	21.69 ± 2.72	22.83 ± 3.73	1.940	0.055
Duration (months)	-	48 (24, 96)	-	-
Number of episodes	-	2 (1, 4)	-	-
PANSS scores				
Positive symptoms	-	20.01 ± 4.82	-	-
Negative symptoms	-	17.99 ± 4.71	-	-
General psychopathology	-	38.25 ± 7.61	-	-
Total	-	83.04 ± 14.38	-	-
BACS				
Verbal memory test (VM)	53.03 ± 8.19	38.80 ± 11.00	7.655	<0.0001
Digit sequencing test (DS)	25.70 ± 1.93	20.78 ± 4.40	7.352	<0.0001
Token motor task (TM)	92.84 ± 8.01	66.86 ± 18.08	9.421	<0.0001
Category fluency (CF)	28.56 ± 7.73	20.58 ± 6.58	6.245	<0.0001
Word fluency (WF)	16.28 ± 4.80	12.06 ± 4.40	4.583	<0.0001
Symbol coding (SC)	69.46 ± 9.00	51.84 ± 16.39	6.875	<0.0001
Tower of London (TL)	19.50 ± 1.74	16.46 ± 4.48	4.593	<0.0001
Total score	0 ± 1	−1.91 ± 0.99	2.465	<0.0001

Note: data are mean ± SD (range) or interquartile range [M (P25, P75)]; *p* < 0.05, the difference was statistically significant. Abbreviations: BMI: body mass index; PANSS: Positive and Negative Syndrome Scale; BACS: Brief Assessment of Cognition in Schizophrenia; HCs: healthy controls; SCZ: schizophrenia.

**Table 2:** Correlation analysis of TRPV1 receptors and some oxidative stress indicators in PBMCs and ADEs with PANSS and BACS in the patient group.

Parameters	PBMCs				ADEs			
	TRPV1	Sirt3	SOD2	acetyl-SOD2	TRPV1	Sirt3	SOD2	acetyl-SOD2
PANSS								
Total score	−0.0263	0.2779	0.2959 *	0.0397	−0.1799	0.0588	−0.0460	0.1371
Positive symptoms	0.3145 *#	−0.4231 **#	0.0031	−0.2458	0.0751	−0.3137 *	−0.0788	0.0505
Negative symptoms	−0.2991 **#	−0.0314	0.2654	0.2822 *#	−0.3255 *#	−0.2643	−0.0331	0.1713
General psychopathology	−0.1151	0.2418	0.3061 *#	0.08187	−0.1961	0.1088	0.0087	0.1436
BACS								
VB	0.5532 ****#	0.0833	−0.1334	−0.3733 **	0.6825 ***#	−0.0943	−0.0505	−0.0641
DS	0.1943	0.2214	0.0140	−0.0622	0.3354 *	−0.0855	−0.0985	0.0033
TM	0.6903 ****#	−0.0194	−0.0566	−0.3794 **#	0.0729	−0.1075	−0.1883	−0.2403
CF	0.6681 ****#	−0.0131	−0.1118	−0.6618 ****#	0.212	0.0922	−0.1669	−0.2969 *
WF	0.5791 ****#	0.0602	−0.0290	−0.4173 **#	0.1972	−0.1590	−0.3218 *	−0.3432 *
SC	0.3038 *	−0.1401	0.2108	−0.278	−0.2011	0.1161	−0.2709	−0.2889 *
TL	0.4753 ***#	0.1696	0.0306	−0.3054 *	−0.0109	0.1191	−0.1914	−0.2252
Total score	0.8658 ****#	0.0447	−0.0551	−0.6401 ****#	0.2929 *	0.0611	−0.3042 *	−0.3986 **#

Note: * *p* < 0.05, ** *p* < 0.01, *** *p* < 0.001, **** *p* < 0.0001; # retains statistical significance following Bonferroni adjustment. r < 0 was negatively correlated, and r > 0 was positively correlated. Abbreviations: PBMC: peripheral blood mononuclear cell; ADEs: astrocyte-derived extracellular vesicles; TRPV1: transient receptor potential vanilloid type 1; PANSS: Positive and Negative Syndrome Scale; BACS: Brief Assessment of Cognition in Schizophrenia; VB: verbal memory test; DS: digit sequencing test; TM: token motor task; CF: category fluency; WF: word fluency; SC: symbol coding; TL: Tower of London.

## Data Availability

The data that support the findings of this study are available from the corresponding author upon reasonable request, as is the study protocol.

## Supplementary Materials

The following supporting information can be downloaded at: https://www.mdpi.com/article/10.3390/brainsci15040339/s1, Table S1: Homogenized expression of TRPV1 and partial oxidative stress indicators in the PBMCs and ADEs; Table S2: Relationship between TRPV1 and partial oxidative stress indicators in the PBMCs and ADEs from patients; Table S3: Relationship between TRPV1 and partial oxidative stress indicators in the PBMCs and ADEs from controls; Table S4: Correlation analysis of TRPV1 and partial oxidative stress indicators in the PBMCs and ADEs with the PANSS and BACS in the patient group; Table S5: Correlation analysis of TRPV1 and some oxidative stress indicators in PBMC and ADEs with BACS in the control group.
