# Supplementary material for: Association of TRPV1 and the SIRT3/SOD2 Signaling Pathway in Mononuclear Cells and Astrocyte-Derived Extracellular Vesicles in Patients with Schizophrenia
Source: Brain Sci. 2025 Mar 25;15(4):339. doi: 10.3390/brainsci15040339 (PMC12025208; doi:10.3390/brainsci15040339)
Supplement: Supplementary file 1 [file brainsci-15-00339-s001.zip › brainsci-3538492-supplementary.pdf]

Supplementary Material

Table S1. Homogenized expression of TRPV1 and partial oxidative stress indicators in the PBMCs and ADEs.

Parameters	HC	SCZ	*t*	*p*
PBMCs				
TRPV1	1.00 ± 0.27	0.63 ± 0.28	6.768	< .0001
Sirt3	1.00 ± 0.27	0.74 ± 0.25	5.018	< .0001
SOD2	1.00 ± 0.20	1.22 ± 0.31	4.263	< .0001
acetyl − SOD2	1.00 ± 0.44	1.73 ± 0.50	7.716	< .0001
Acetyl − SOD2/SOD2	1.00 ± 0.46	1.44 ± 0.56	4.334	< .0001
ADEs				
TRPV1	1.00 ± 0.20	0.73 ± 0.19	6.789	< .0001
Sirt3	1.00 ± 0.27	0.71 ± 0.18	6.363	< .0001
SOD2	1.00 ± 0.17	1.24 ± 0.23	5.860	< .0001
acetyl − SOD2	1.00 ± 0.29	1.86 ± 0.32	14.10	< .0001
acetyl − SOD2/SOD2	1.00 ± 0.28	1.53 ± 0.30	9.014	< .0001

Note: data are mean ± SD (range); $p < 0.05$, the difference was statistically significant. Abbreviations: PBMC: peripheral blood mononuclear cell; ADEs: astrocyte − derived extracellular vesicles; TRPV1: transient receptor potential vanilloid type 1; HC: healthy control; SCZ: schizophrenia.

Table S2. Relationship between TRPV1 and partial oxidative stress indicators in the PBMCs and ADEs from patients.

Parameters			PBMCs				ADEs			
			**TRPV1**	**Sirt3**	**SOD2**	**acetyl－SOD2**	**TRPV1**	**Sirt3**	**SOD2**	**acetyl－SOD2**
PBMCs	TRPV1	*r*	–	–	–	–	–	–	–	–
		p	–	–	–	–	–	–	–	–
	Sirt3	*r*	0.0723	–	–	–	–	–	–	–
		p	0.6176	–	–	–	–	–	–	–
	SOD2	*r*	− 0.0844	0.0417	–	–	–	–	–	–
		p	0.5599	0.7735	–	–	–	–	–	–
	acetyl－SOD2	*r*	− 0.6917	− 0.0336	0.2863	–	–	–	–	–
		p	< .001	0.8167	< .05	–	–	–	–	–
ADEs	TRPV1	*r*	0.2882	0.1844	− 0.1517	− 0.1347	–	–	–	–
		p	< .05	0.1998	0.2930	0.3515	–	–	–	–
	Sirt3	*r*	0.1613	− 0.0148	− 0.0257	− 0.1615	0.2485	–	–	–
		p	0.2630	0.9187	0.8596	0.2627	0.0818	–	–	–
	SOD2	*r*	− 0.3258	− 0.0548	0.0194	0.3540	0.1287	0.2637	–	–
		p	< 0.05	0.7056	0.8936	< .05	0.3736	0.0642	–	–
	acetyl－SOD2	*r*	− 0.4378	0.1176	0.1724	0.5729	0.1360	− 0.0316	0.4316	–
		p	< .01	0.4146	0.2313	< .001	0.3465	0.8271	< .01	–

Note: $p < 0.05$, the difference was statistically significant; $r < 0$ was negatively correlated, and $r > 0$ was positively correlated; － indicates repetition or nonsense.

Abbreviations: PBMC: peripheral blood mononuclear cell; ADEs: astrocyte－derived extracellular vesicles; TRPV1: transient receptor potential vanilloid type 1.

Table S3. Relationship between TRPV1 and partial oxidative stress indicators in the PBMCs and ADEs from controls.

Parameters			PBMCs				ADEs			
			**TRPV1**	**Sirt3**	**SOD2**	**acetyl − SOD2**	**TRPV1**	**Sirt3**	**SOD2**	**acetyl − SOD2**
PBMCs	TRPV1	*r*	–	–	–	–	–	–	–	–
		p	–	–	–	–	–	–	–	–
	Sirt3	*r*	0.0894	–	–	–	–	–	–	–
		p	0.5369	–	–	–	–	–	–	–
	SOD2	*r*	0.0504	− 0.0280	–	–	–	–	–	–
		p	0.7281	0.8471	–	–	–	–	–	–
	acetyl − SOD2	*r*	− 0.1204	− 0.0478	0.1712	–	–	–	–	–
		p	0.4051	0.7418	< .05	–	–	–	–	–
ADEs	TRPV1	*r*	0.1389	− 0.0678	− 0.0682	− 0.0071	–	–	–	–
		p	0.3360	0.6398	0.6381	0.0996	–	–	–	–
	Sirt3	*r*	0.1814	0.0054	− 0.2171	− 0.2130	0.0723	–	–	–
		p	0.2074	0.9701	0.1298	0.1375	0.6176	–	–	–
	SOD2	*r*	− 0.0883	− 0.1482	0.0450	0.1713	− 0.0844	0.0417	–	–
		p	0.5420	0.3043	0.7563	0.0793	0.5599	0.7735	–	–
	acetyl − SOD2	*r*	− 0.1851	− 0.0613	0.1606	0.0523	− 0.6917	− 0.0336	0.2863	–
		p	0.1980	0.3723	0.2652	0.0971	0.0657	0.1167	< .05	–

Note: $p < 0.05$, the difference was statistically significant; $r < 0$ was negatively correlated, and $r > 0$ was positively correlated; − indicates repetition or nonsense.

Abbreviations: PBMC: peripheral blood mononuclear cell; ADEs: astrocyte − derived extracellular vesicles; TRPV1: transient receptor potential vanilloid type 1.

Table S4. Correlation analysis of TRPV1 and partial oxidative stress indicators in the PBMCs and ADEs with the PANSS and BACS in the patient group (*p* value).

Parameters	PBMC				ADEs			
	TRPV1	Sirt3	SOD2	acetyl − SOD2	TRPV1	Sirt3	SOD2	acetyl − SOD2
PANSS								
Total score	0.8562	0.0507	< .05	0.7843	0.2112	0.6849	0.7510	0.3424
Positive symptoms	< .05	< .01	0.9831	0.0853	0.6041	< .05	0.5864	0.7278
Negative symptoms	< .01	0.8287	0.0635	< .05	< .05	0.0636	0.8195	0.2342
General psychopathology	0.4262	0.0907	< .05	0.5719	0.1722	0.4518	0.9522	0.3198
BACS								
VB	< .0001	0.5652	0.3556	< .01	< .001	< .05	0.7279	0.6582
DS	0.1763	0.1222	0.9230	0.6676	< .05	0.5546	0.4963	0.9821
TM	< .0001	0.8938	0.6964	< .01	0.6147	0.4574	0.1904	0.0928
CF	< .0001	0.9281	0.4394	< .0001	0.1395	0.5243	0.2467	< .05
WF	< .0001	0.6780	0.8415	< .01	0.1700	0.2700	< .05	< .05
SC	< .05	0.3318	0.1418	0.0506	0.1615	0.4220	0.0571	< .05
TL	< .001	0.2389	0.8331	< .05	0.9401	0.4102	0.1830	0.1159
Total score	< .0001	0.0477	0.0551	< .0001	< .05	0.0611	< .05	< .01

Note: Abbreviations: PANSS: Positive and Negative Syndrome Scale; BACS: Brief Assessment of Cognition in Schizophrenia; PBMC: peripheral blood mononuclear cell; ADEs: astrocyte − derived extracellular vesicles; TRPV1: transient receptor potential vanilloid type 1; VB: verbal memory test; DS: digit sequencing test; TM: token motor task; CF: category fluency; WF: word fluency; SC: symbol coding; TL:

Tower of London. $p < 0.05$, the difference was statistically significant.

Table S5. Correlation analysis of TRPV1 and some oxidative stress indicators in PBMC and ADEs with BACS in the control group.

Parameters		PBMC				ADEs			
		TRPV1	Sirt3	SOD2	acetyl − SOD2	TRPV1	Sirt3	SOD2	acetyl − SOD2
VB	*r*	0.5772	0.2857	− 0.0185	0.092	0.5733	0.4074	− 0.2770	− 0.3821
	p	< .0001	0.0643	0.8987	0.5251	< .0001	0.0533	0.1129	< .01
DS	*r*	0.1327	− 0.086	− 0.1498	− 0.2438	0.1469	− 0.0958	− 0.1402	− 0.3707
	p	0.3582	0.5526	0.2991	0.0880	0.3087	0.5083	0.3314	0.0803
TM	*r*	0.4185	− 0.0344	− 0.1913	− 0.1434	0.5154	0.4704	− 0.4096	− 0.3943
	p	< .01	0.8121	0.1833	0.3204	< .001	0.0601	0.0531	< .01
CF	*r*	0.2507	0.2604	− 0.0001	− 0.0627	0.2363	0.2072	− 0.3935	− 0.1306
	p	0.0791	0.0678	0.9994	0.6650	0.0987	0.1488	0.0747	0.3658
WF	*r*	0.2132	0.0156	0.0534	− 0.2201	0.2719	0.0922	0.0083	− 0.0218
	p	0.1372	0.9146	0.7124	0.1245	0.0561	0.2407	0.9545	0.8807
SC	*r*	0.3306	0.1529	0.01372	0.2069	0.5915	0.3163	− 0.0407	− 0.3016
	p	< .05	0.2891	0.9247	0.1493	< .0001	0.0522	0.7789	0.0533
TL	*r*	0.3803	0.1287	− 0.2756	− 0.1845	0.4109	0.3694	− 0.1965	− 0.3022
	p	< .01	0.3729	0.0527	0.1996	< .01	0.0803	0.1713	< .05
Total score	*r*	0.5921	0.1572	− 0.352	− 0.015	0.6396	0.1438	− 0.1498	− 0.4153
	p	< .01	0.2756	0.0712	0.6179	< .001	0.3191	0.2991	< .05

Note: Abbreviations: BACS: Brief Assessment of Cognition in Schizophrenia; PBMC: peripheral blood mononuclear cell; ADEs: astrocyte − derived extracellular vesicles; TRPV1: transient receptor potential vanilloid type 1; VB: verbal memory test; DS:

digit sequencing test; TM: token motor task; CF: category fluency; WF: word fluency; SC: symbol coding; TL: Tower of London. $r < 0$ was negatively correlated, and $r > 0$ was positively correlated; $p < 0.05$, the difference was statistically significant.
